# Secondary metabolites from *Bacillus amyloliquefaciens* isolated from soil can kill *Burkholderia pseudomallei*

**DOI:** 10.1186/s13568-016-0302-0

**Published:** 2017-01-04

**Authors:** Patcharaporn Boottanun, Chotima Potisap, Julian G. Hurdle, Rasana W. Sermswan

**Affiliations:** 1 Department of Biochemistry, Faculty of Medicine, Khon Kaen University, 123 Mitraparb Rd, Muang District, Khon Kaen Province 40002 Thailand; 2 Melioidosis Research Centre, Khon Kaen University, 123 Mitraparb Rd, Muang District, Khon Kaen Province 40002 Thailand; 3 Center for Infectious and Inflammatory diseases, Institute of Biosciences and Technology, Texas A&M University, 2121W. Holcombe Blvd, Houston, TX 77030 USA

**Keywords:** Bio-control, Antimicrobial peptides, Pathogenic bacteria, Secondary metabolites

## Abstract

*Bacillus* species are Gram-positive bacteria found in abundance in nature and their secondary metabolites were found to possess various potential activities, notably antimicrobial. In this study, *Bacillus amyloliquefaciens* N2-4 and N3-8 were isolated from soil and their metabolites could kill *Burkholderia pseudomallei,* a Gram-negative pathogenic bacterium also found in soil in its endemic areas. Moreover, the metabolites were able to kill drug resistant isolates of *B. pseudomallei* and also inhibit other pathogenic bacteria such as *Staphylococcus aureus*, *Escherichia coli* and *Acinetobacter baumannii* but not the non-pathogenic *Burkholderia thailandensis*, which is closely related to *B. pseudomallei*. Since the antimicrobial activity of N3-8 was not partially decreased or abolished when treated with proteolytic enzymes or autoclaved, but N2-4 was, these two strains should have produced different compounds. The N3-8 metabolites with antimicrobial activity consisted of both protein and non-protein compounds. The inhibition spectrum of the precipitated proteins compared to the culture supernatant indicated a possible synergistic effect of the non-protein and peptide compounds of N3-8 isolates against other pathogens. When either N2-4 or N3-8 isolates was co-cultured with *B. pseudomallei* the numbers of the bacteria decreased by 5 log_10_ within 72 h. Further purification and characterization of the metabolites is required for future use of the bacteria or their metabolites as biological controls of *B. pseudomallei* in the environment or for development as new drugs for problematic pathogenic bacteria.

**Electronic supplementary material:**

The online version of this article (doi:10.1186/s13568-016-0302-0) contains supplementary material, which is available to authorized users.

## Introduction

*Bacillus* spp. are Gram-positive bacteria found diversely in nature especially in soil. In unsuitable conditions such as high temperature, radiation and harsh chemical reagents, they can form endospores for survival (Errington 2003). Besides spore forming, *Bacillus* spp. are also able to produce secondary metabolite products (Sansinenea and Ortiz 2011), which is an additional function to compete against other organisms. Bioactive compounds from *Bacillus subtilis,* bacteriocin-like substances, were reported to inhibit several clinical bacteria such as *Listeria monocytogenes*, *Staphylococcus aureus, Bacillus cereus*, *Salmonella typhi* (Xie et al. 2009) and also a substance from *Bacillus licheniformis* could inhibit food spoilage bacteria (Guo et al. 2012). Moreover, the culture supernatant from *Bacillus* spp. isolated from soil named KW and SA was reported to contain N-acyl homoserine lactone that significantly decreased biofilm formation of *Burkholderia pseudomallei* (Ramli et al. 2012). In addition, the *Bacillus* strain TKS1-1 in endospore form, was used to reduce the incidence of citrus bacterial canker (Huang et al. 2012). Some *Bacillus* spp. can produce several types of active compounds such as *B. amyloliquefaciens* FZB42 that has 8.5% of the genome dedicated for the synthesis of secondary metabolites (Chen et al. 2007). It can produce lipopeptides; surfactin, fengycin, bacillomycin D, polyketide (difficidin) and dipeptide bacilysin that can suppress growth of *Erwinia amylovora* (Chen et al. 2009).

Melioidosis is one of infectious diseases that pose a significant public health problem in Southeast Asia and North Australia. The causative agent is *B. pseudomallei,* a Gram-negative bacteria which can be found in soil and water in endemic areas (Cheng and Currie 2005). Melioidosis is the third most cause of death among the infectious diseases in northeast Thailand and is responsible for 20% of community acquired septicemias with a 40% mortality rate (Wiersinga et al. 2012). The bacterium is intrinsically resistant to several antibiotics and acquired resistant to ceftazidime the drug of choice after lost a penicillin-binding protein 3 gene (Chantratita et al. 2011). *Burkholderia pseudomallei* was found to be unevenly distributed in soil and that the physicochemical properties of soil may partially influence the presence and absence of the bacterium (Ngamsang et al. 2015; Palasatien et al. 2008). Some microbes in soil may also inhibit or compete by producing some active compounds against the bacteria.

In this study, it was therefore of interest to screen for *Bacillus* spp. in soils that were negative for *B. pseudomallei* and characterize the metabolites that could inhibit or kill *B. pseudomallei*. The *Bacillus* spp. themselves or their antimicrobial metabolites might then be used as bio-controls to prevent and reduce the incidence of melioidosis in endemic areas.

## Materials and methods

### Bacterial strains

Bacterial strains used to test for the inhibition spectrum of *B. amyloliquefaciens* metabolites are listed in Additional file 1: Table S1. They were obtained from the Melioidosis Research Center, Faculty of Medicine Khon Kaen University, Thailand and also kindly provided by Associate Professor Julian G. Hurdle’s laboratory, Center for Infectious and Inflammatory diseases, Institute of Biosciences and Technology, Texas A&M University, USA. Our bacterial strains were deposited in culture collection belonging to World Data Centre for Microorganism (WDCM) as MRCKKU (registration number 1130).

### Isolation of *Bacillus* spp. from soil

Twenty-five soil samples that were confirmed as negative for *B. pseudomallei* by culture and semi**-**nested PCR (Ngamsang et al. 2015) were used for isolation of *Bacillus* spp.

The protocol for isolation of *Bacillus* spp. from soil was according to Travers et al. (1987) described with some modifications. One gram of each soil sample was mixed with 10 ml sterile distilled water and boiled at 100 °C for 5 min to kill other vegetative cells. Thereafter, supernatants were diluted by 10-fold serial dilution and 100 µl of the 10^−2^, 10^−3^, 10^−4^ dilutions were spread onto nutrient agar (NA) plates and incubated at 37 °C for 18–24 h. Bacterial isolates with colony morphology of large, dry, white color with wavy, lobed margins were selected to sub-culture and confirmed by the Gram’s stain. These isolates were tested for their ability to kill *B. pseudomallei* and other pathogens.

### Agar well diffusion method for screening of antimicrobial activity

Antimicrobial activity against *B. pseudomallei* or other pathogens of culture supernatants and precipitated protein from culture supernatants of *Bacillus* spp. were investigated by the agar well diffusion method (Umer et al. 2013). In brief, overnight 1% cultures in Luria Bertani (LB) medium of *B. pseudomallei* and other indicator bacteria were inoculated into fresh LB medium and incubated at 37 °C, 200 rpm for 4 h until the log phase and were then used at approximately 10^5^–10^6^ CFU/ml to swab on Müller-Hinton agar (MHA) plates. The plates were punched to obtain 6.6 mm wells by sterile pipette tips and then 100 µl of sterile supernatant or precipitated proteins were added into each well. Ceftazidime (Sigma**-**Aldrich, St. Loius, MO, USA) at 50 µg/ml concentration was used as the positive control and minimal medium was used as the negative control. The plates were left at room temperature for 1 h before being incubated at 37 °C, for 18–24 h. Inhibition activity was evaluated by measuring the diameter of inhibition zone against *B. pseudomallei*.

### Species identification

N2-4 and N3-8 with inhibitory activity against *B. pseudomallei* were selected for gDNA extraction with an RBC kit (RBC ribosicen, Taiwan) and were then used for PCR amplification using universal primers against the conserved region in 16s rDNA gene (Rd1; 5′AAGGAGGTGATCCAGCC3′, Fd1; 5′AGTTTGATCCTGGCTCAG3′) (Ghribi et al. 2012). The master mix of PCR contained 2.5 µl of 10X PCR buffer, 0.16 mM dNTP, 2.0 mM MgCl_2_, 0.1 µM of each primer, 0.04 unit/ml of Taq DNA polymerase, 50 ng of DNA template and DNase**-**free water to a final volume of 25 µl. The PCR products were analyzed with 1.2% agarose gel electrophoresis and stained with SYRB Gold. The expected PCR products of 1500 bps were sequenced (First Base laboratories Sdn Bhd, Malaysia) and the BLASTn program was used (Altschul et al. 1997; National Center for Biotechnology Information 2015) to identify their species.

### Production kinetics of antimicrobial metabolites

N2-4 and N3-8 were cultured in duplicate in 200 ml of minimal medium composed of 5.0 g l-glutamic acid, 0.5 g KH_2_PO_4_, 0.5 g K_2_HPO_4_, 0.2 g MgSO_4_·7H_2_O, 0.01 g MnSO_4_·H_2_O, NaCl 0.01 g, FeSO_4_·7H_2_O 0.01 g, CuSO_4_·7H_2_O 0.01 g and CaCl_2_·2H_2_O 0.015 g/L. This was supplemented with 1% w/v sterile glucose as described by Jamil et al. (2007) at 37 °C with 200 rpm shaking for 102 h. Every 6 h, 1 ml of the culture was sampled to obtain the culture supernatant for antimicrobial activity to test against *B. pseudomallei* using the agar well diffusion method.

### Partial characterization of antimicrobial metabolites from N2-4 and N3-8

#### Proteolytic enzymes susceptibility test

The culture supernatants from N2-4 and N3-8 were treated with 200 µg/ml of proteinase K (Amresco, Solon, OH, USA) and 1 mg/ml of pepsin, trypsin and papain (Sigma**-**Aldrich, St. Loius, MO, USA) at final concentrations and incubated at 37 °C for 3 h. For inactivation, the proteinase K was processed at 75 °C for 10 min or 100 °C for 5 min for the other three enzymes to determine the antimicrobial activity against *B. pseudomallei* by the agar wells diffusion method.

#### Thermo stability test

The culture supernatants from N2-4 and N3-8 were incubated at 25, 37, 40, 50, 60, 70, 80, 90, 100, and 121 °C for 15 min and then tested for the antimicrobial activity against *B. pseudomallei* by the agar well diffusion method.

#### Antimicrobial spectrum

For a quick screening of the antimicrobial activity, the cross steak method was used as described by Hemashenpagam with some modifications (Hemashenpagam 2011). N2-4 or N3-8 isolates were streaked as a line at the center of the NA plate and incubated for 72 h. After that, the overnight culture of indicator bacteria (Additional file 1: Table S1) was streaked across the single streak of N2-4 and N3-8 isolates and incubated at 37 °C for 24 h. The qualitative grading of inhibition assigned ++++ for the highest, +++ for moderate, ++ for a few, + for low activity and − for no inhibition.

### Production of proteins from N2-4 and N3-8

The production medium of secondary metabolites is a minimal medium supplemented with 1% w/v glucose sterile with a filtered 0.2 µm syringe (Jamil et al. 2007). One percent of both *B. amyloliquefaciens* N2-4 and N3-8 were inoculated into minimal media and kept at 37 °C, 200 rpm for 72 h. The cultured supernatants were harvested by centrifugation at 16,000×*g* for 15 min, (Avanti^®^ J**-**E, Beckman Coulter, CA, USA) and then filtered through a 0.2 µm membrane. These crude metabolites were used for protein purification or kept at −20 °C until used for the inhibition assay.

### Partial purification of proteins

Culture supernatants from N2-4 and N3-8 were precipitated using 20, 40, 60 and 80% saturated ammonium sulfate ((NH_4_)_2_SO_4_) as described by Sharma with some modification (Sharma et al. 2009). The precipitated proteins were harvested by centrifugation at 13,000×*g* (Avanti^®^ J**-**E, Beckman Coulter, CA, USA) at 4 °C for 20 min. The protein pellets were then re**-**suspended in TE buffer pH 8.0 (10 mM Tris**–**HCl, 1 mM EDTA) and dialyzed with similar buffers using a dialysis bag with a 3.5 kDa cut off (Thermo scientific, Rockford, IL, USA) at 4 °C overnight. The activity of these precipitated proteins was confirmed against *B. pseudomallei* and other pathogenic bacteria by the agar well diffusion method. The concentrations of the precipitated proteins were measured by the Bradford technique (Biorad, CA, USA) according to the manufacturers’ protocol.

The precipitated proteins were prepared to have a concentration of 3.0 mg/ml and were tested for antimicrobial activity against 14 pathogenic bacteria by the agar well diffusion method as listed in the Additional file 1: Table S1.

### Time-kill assay of the precipitated proteins

The time-kill assay, as described by Sopirala et al. (2010), with some modifications, was used to determine the time that *B. pseudomallei* was killed after N2-4 and N3-8 precipitated proteins were added into the *B. pseudomallei* cultures. Briefly, 3.0 mg/ml of precipitated proteins were twofold serial diluted with MHB in 24-well plates and then added 10^5^–10^6^ CFU/ml of *B. pseudomallei* and incubated at 37 °C. After incubation for 3, 6, 12 and 24 h, 100 µl from each dilution was 10-fold serially diluted with PBS pH 7.2 and 10 µl of each dilution were used to drop on selective Ashdown’s agar (Naghili et al. 2013) for *B. pseudomallei* colony counts.

### Minimum inhibitory concentration (MIC) and Minimum bactericidal concentration (MBC) of antimicrobial compounds

The precipitated proteins from N2-4 and N3-8 isolates were filtered through 0.2 µm membranes and the concentrations adjusted to 3.0 mg/ml and used to determine the MIC and MBC by micro-broth dilution (Hoelzer et al. 2011). In brief, the antimicrobial compounds were diluted in 96-well plates by twofold serial dilutions using MHB. Then *B. pseudomallei* of approximately 10^5^–10^6^ CFU/ml was added into each well, mixed gently and then incubated at 37 °C for 18**–**24 h. The last concentration that provided clear solution when compared to the growth control was recorded as the MIC. The MBC was evaluated by pipette each dilution from the clear wells, diluting with PBS pH 7.2 and then 10 µl of each dilution was dropped onto Ashdown’s agar for colony counts. The interpretation was that the MBC must have decreased ≥3 log_10_ CFU/ml or 99.9% of the bacterial cell count when compared to growth control.

### Identification of proteins with antimicrobial activity

The precipitated proteins were separated by native-PAGE in the tris–glycine system and the separated bands were used to test for the inhibitory activity as described by Barbaza-Corana (2007) with some modifications. Precipitated proteins were separated in duplicate; one strip was washed with sterile deionized water and placed on the MHA plate that was spread with 10^5^ **–**10^6^ CFU/ml *B. pseudomallei,* the strip without proteins loading was used as negative control. The inhibition zone against *B. pseudomallei* that was caused by proteins was observed after incubation at 37 °C for 18–24 h. Another strip of this gel was stained with silver stain (Schägger 2006) to determine the molecular weight of the protein band that showed inhibition activity.

### Bio-control of *B. pseudomallei* using *B. amyloliquefaciens*

*B. pseudomallei* at 6 × 10^6^ CFU/ml were co-cultured with 6 × 10^4^ CFU/ml of N2-4 or N3-8 isolates in 100 ml of LB medium in 250 ml Erlenmeyer flasks and incubated at 37 °C with 200 rpm shaking. The experiment was done in triplicate. *Burkholderia pseudomallei* cultured in the same condition was used as a control. The viability of *B. pseudomallei* was measured after co-culture for 24, 48, 72 and 96 h using the plate count method on Ashdown’s agar.

## Results

### Bacterial isolation and species identification

**Fig. 1:**
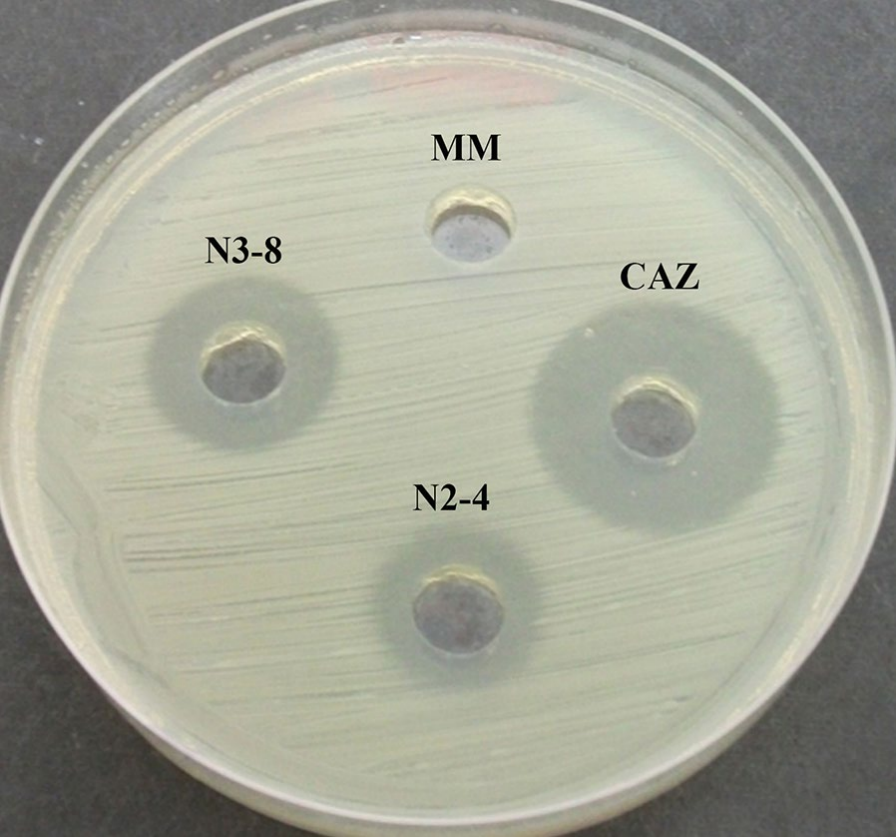
The antimicrobial activity of culture supernatants from *B. amyloliquefaciens* N2-4 and N3-8 isolates against *B. pseudomallei*. The inhibitory activity of culture supernatants from *B. amyloliquefaciens* N2-4 and N3-8 isolates against *B. pseudomallei* by the agar well diffusion method as seen by clear zones, *MM* minimal medium (negative control), *CAZ* ceftazidime 50 µg/mL, the drug of choice for *B. pseudomallei* (positive control)

Sixty-six isolates of Gram-positive bacilli, with morphology of being large, dry, white colored, with wavy, lobed margins were obtained. After the antimicrobial activity against *B. pseudomallei* was observed by the agar well diffusion method, the two isolates named N2-4 and N3-8 showed clear zones of inhibition (Fig. 1). PCR amplification against the conserved regions in 16s rDNA genes of N2-4 and N3-8 gave the expected 1500 bps products. The nucleotide sequences shown by BLASTn indicated 99% similarity to *B. amyloliquefaciens*. The accession numbers of 16 s rDNA that were identical to 16 s rDNA of N2-4 and N3-8 are KC887505.1 and HG32825.1.

### Production time of antimicrobial metabolites

**Fig. 2:**
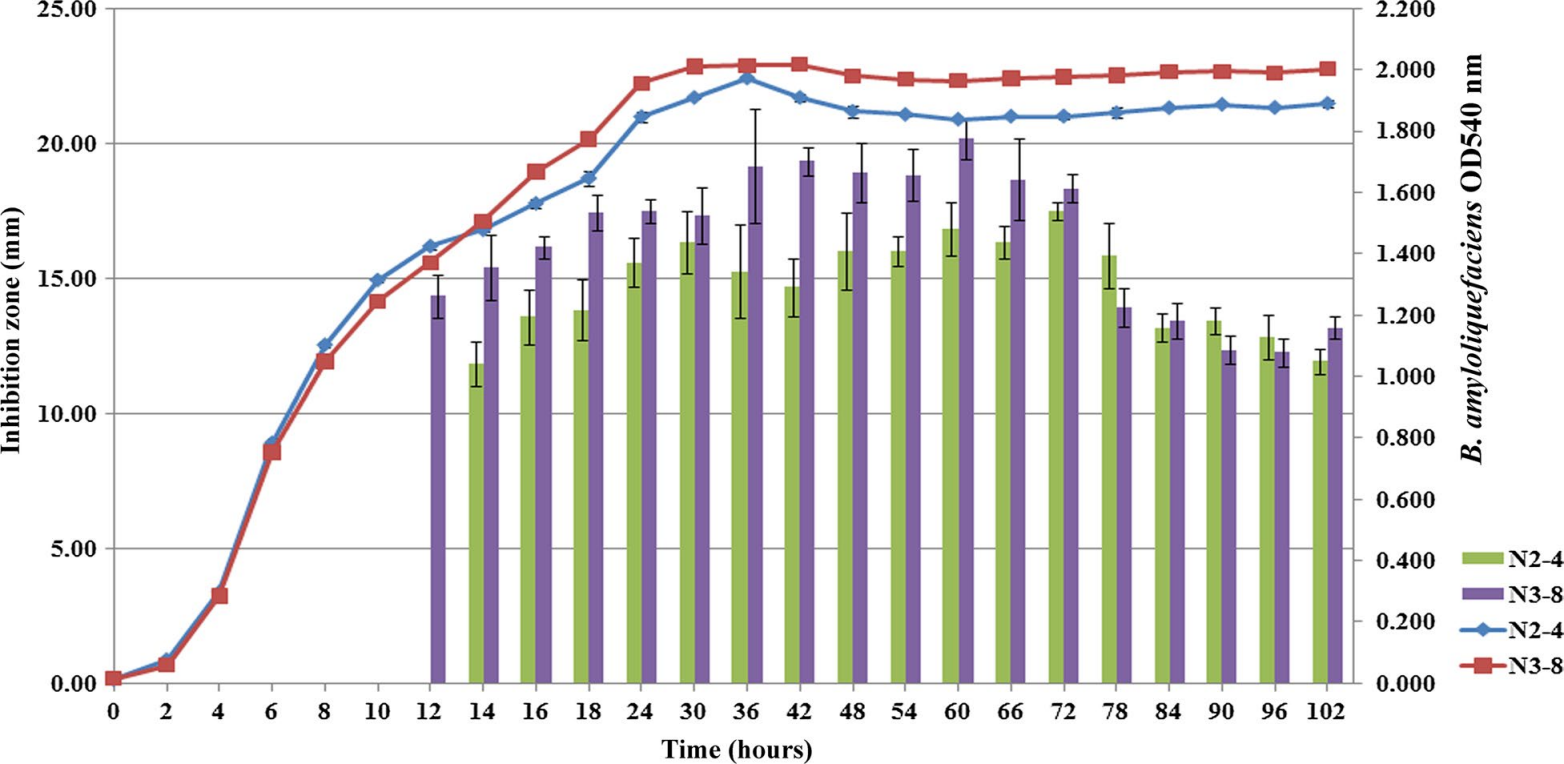
The production of secondary metabolites from *B. amyloliquefaciens* N2-4 and N3-8 isolates. The production of secondary metabolites displayed as sizes of the inhibition zones in nm against *B. pseudomallei* as evaluated by the agar well diffusion method from N2-4 (*green bars*) and N3-8 (*purple bars*) were plotted on the left Y axis and growth curve as measured at OD540 nm of N2-4 (*blue line*) and N3-8 (*red line*) were plotted on the right Y axis, while X axis represent the time in hours

The metabolites from *B. amyloliquefaciens* N3**-**8 showed more broadened antimicrobial activity against *B. pseudomallei* and was therefore selected to grow in minimum medium in duplicate to observe the production of antimicrobial metabolites in time by the agar well diffusion method. After being cultured for 12 and 14 h, the culture supernatants from N3-8 and N2-4 started to show inhibition against *B. pseudomallei*. The highest antimicrobial activity was observed at 60**–**72 h with the inhibition zone of 20 and 17 mm (Fig. 2).

### Partial characterization of antimicrobial metabolites from *B. amyloliquefaciens*

#### Proteolytic digestion and temperature stability

When the culture supernatants from N3-8 were heated from 25 °C to 121 °C for 15 min, the activity against *B. pseudomallei* decreased at 40–100 °C and was almost halved when autoclaved at 121 °C while metabolites from N2-4 showed decreased in activity after heating at 80–100 °C and were abolished by being autoclaved (Additional file 1: Table S2). The activities of the culture supernatants from N3-8 were partially decreased when treated with all proteolytic enzymes while those of N2-4 were abolished by proteinase K and trypsin and partially decreased by papain and pepsin digestion (Additional file 1: Table S3).

#### Antimicrobial spectrum of N2-4 and N3-8

The culture supernatants from N3-8 inhibited 100% of *B. pseudomallei* clinically sourced strains, 83% of environmentally sourced strains, 100% ceftazidime resistant and 100% in mutant isolates while N2-4 inhibited these same strains at 87, 67, 80 and 60% (Table 1). They did not inhibit all 12 isolates of *B. thailandensis*, a non-pathogenic bacterium that is closely related to *B. pseudomallei*. Using the cross streak method, N2-4 and N3-8 isolates had clear inhibition activity against Gram-positive bacteria such as *S. aureus*, *Clostridium difficile* and *Enterococcus faecium* and other Gram-negative bacteria such as *B. pseudomallei, Acinetobacter baumannii, Pseudomonas aeruginosa* and *E. coli* (Additional file 1: Table S4).

**Table 1:** The antimicrobial activity of culture supernatant from *B. amyloliquefaciens* N2**-**4 and N3**-**8 isolates against *B. pseudomallei* and *B. thailandensis*

Bacterial indicators	*B. amyloliquefaciens* N2-4 supernatants	*B. amyloliquefaciens* N 3-8 supernatants
*B. pseudomallei* clinical isolates (16)	14	16
*B. pseudomallei* environmental isolates (6)	4	5
*B. pseudomallei* CAZ resistance strains (5)	4	5
*B. pseudomallei* mutant strains (5)	3	5
M6 biofilm mutant	−	+
M10 biofilm mutant	+	+
SRM117 LPS O-side chain mutant	−	+
MM35 flagellin mutant	+	+
SR1015 capsule mutant	+	+
*B. thailandensis* (12)	0	0

No. in brackets indicated number of test isolates

− Not inhibited, + inhibited

### Partial purification and antimicrobial activity of precipitated proteins

Sixty percent of saturated ammonium sulfate gave the precipitated proteins from the culture supernatants of N2-4 and N3-8 with the highest inhibition activity against *B. pseudomallei.* When these precipitated proteins from N2-4 and N3-8 were tested against Gram-positive and Gram**-**negative bacteria, they effectively inhibited *S. pyogenes, S. pneumonia* and *Moraxella catarrhalis* as shown with very large clear zones of inhibition (Table 2). Precipitated proteins from N3-8, in general, showed larger clear zones than N2-4.

**Table 2:** The antimicrobial activity of precipitated proteins from *B. amyloliquefaciens* N2-4 and N3-8 against pathogenic bacteria

Bacterial indicators	Inhibition zone (Ø mm)	
	*B. amyloliquefaciens* N2-4 proteins	*B. amyloliquefaciens* N3-8 proteins
Gram-positive pathogenic bacteria		
*Corynebacterium diphtheriae*	17	23
*Streptococcus pneumoniae*	>30	>30
*Streptococcus pyogenes*	27	27
Gram-negative pathogenic bacteria		
*B. pseudomallei* p37	21	24
*Moraxella catarrhalis*	>30	>30
*Citrobacter freundii*	14	18.5
*Salmonella* group D	14.5	23
*Proteus vulgaris*	14.5	23
*Escherichia coli*	11	19.5
*Shigella* group D	18	24
*Klebsiella pneumoniae*	11.5	15.5
*Acinetobacter baumannii*	17.6	23
*Vibrio parahaemolyticus*	0	15
*Stenotrophomonas maltophilia*	23.8	28

The MICs of precipitated proteins from N2-4 and N3-8 were 0.19 and 0.02 mg/ml. For MBCs, the concentrations of precipitated proteins from N2-4 and N3-8 that decreased *B. pseudomallei* colony counts by 99% were 0.75 and 0.04 mg/ml.

### Time-kill assay of precipitated proteins

**Fig. 3:**
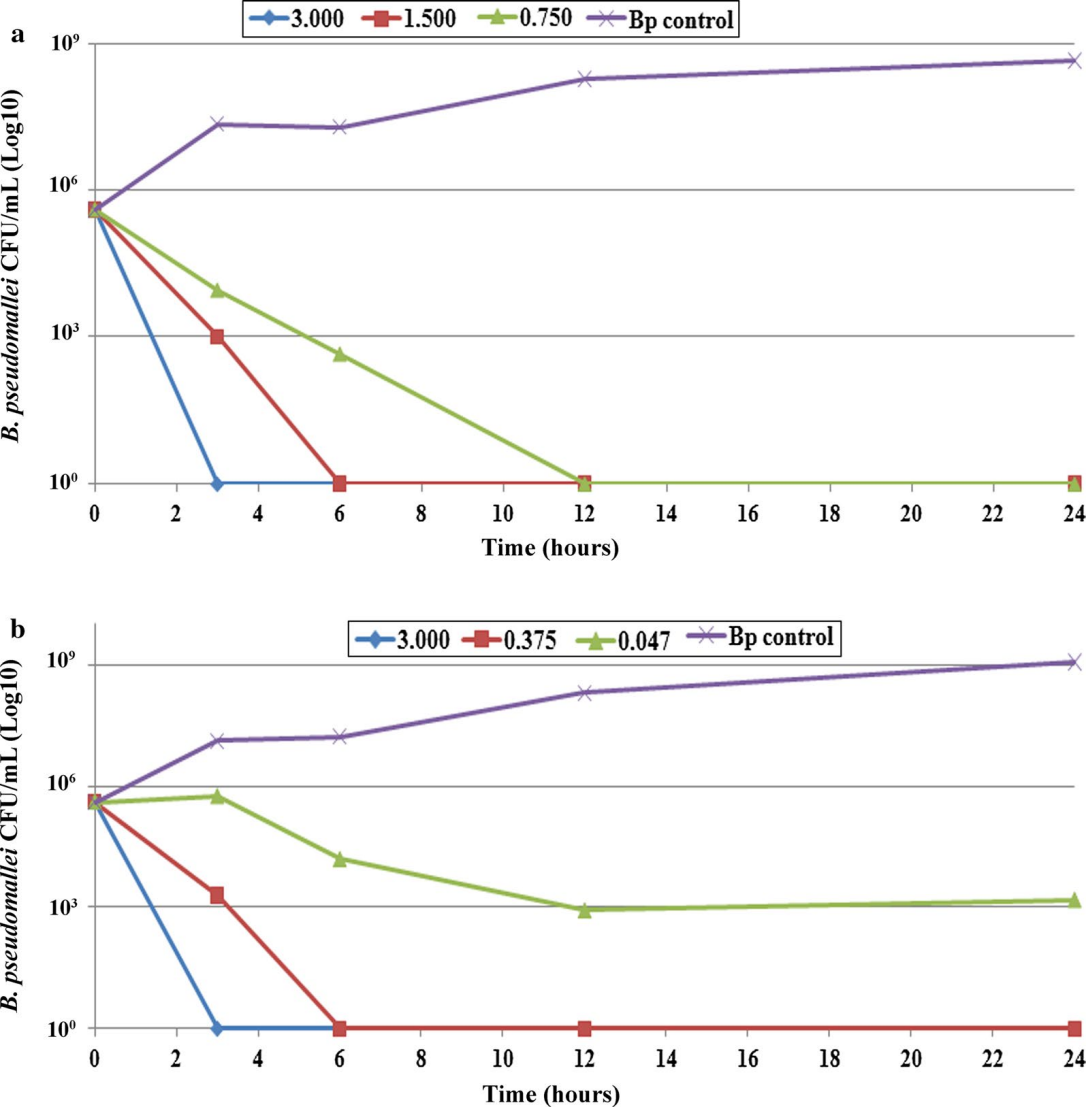
Time-kill assay of precipitated proteins from *B. amyloliquefaciens* against *B. pseudomallei.* **a** Concentrations of precipitated proteins with the MBCs against *B. pseudomallei* from *B. amyloliquefaciens* N2-4 were used to lyse *B. pseudomallei.* The X axis indicates time in hours after various concentrations of the proteins as indicated (mg/mL) were added into *B. pseudomallei* cultures and the Y axis indicates the CFU/ml of *B. pseudomallei* after treatment. **b** Proteins from *B. amyloliquefaciens* N3-8 were tested in the same manner

The results from MBC assay confirmed that precipitated proteins from N2-4 and N3-8 could kill *B. pseudomallei*. The N2-4 precipitated proteins at concentrations of 3.0, 1.5 and 0.75 mg/ml could kill *B. pseudomallei* within 3, 6 and 12 h (Fig. 3a). N3-8 proteins of 3.0 mg/ml could kill *B. pseudomallei* within 3 h and 0.09**-**1.5 mg/ml killed *B. pseudomallei* in 6 h and 0.04 mg/ml killed *B. pseudomallei* after being treated for 12 h (Fig. 3b). Therefore, the killing activity of these active compounds was in a dose dependent manner.

### Identification of proteins with antimicrobial activity

**Fig. 4:**
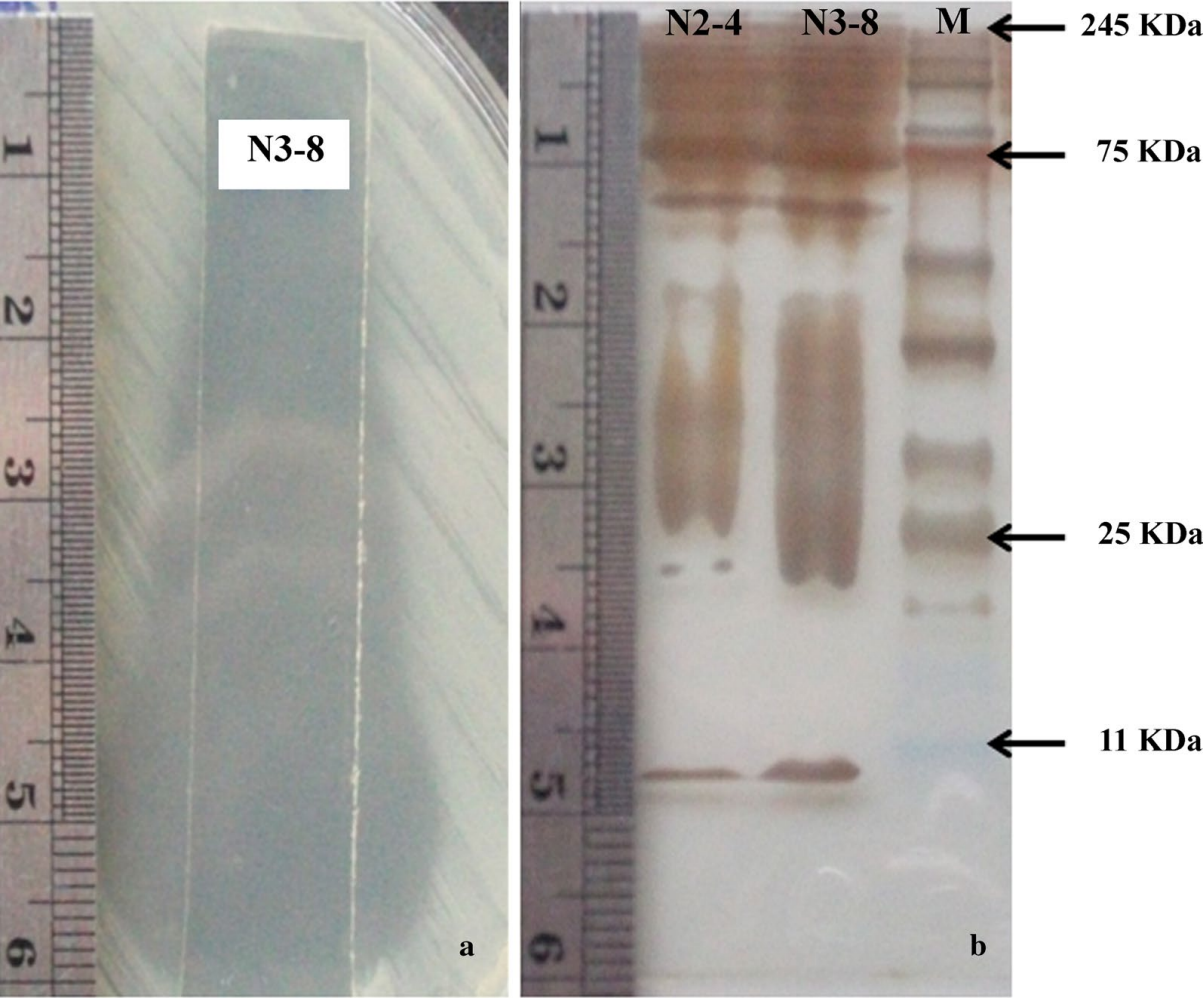
Antimicrobial activity of precipitated proteins from *B. amyloliquefaciens* N2-4 and N3-8 isolates in native-PAGE against *B. pseudomallei*. **a** Clear inhibition zones of precipitated proteins from a strip of native-PAGE that was placed on the *B. pseudomallei* lawn. **b** Precipitated proteins from N2-4 and 3-8 were separated by native-PAGE and visualized by silver staining. *M* represents the size marker

Following the results of the in**-**gel overlay assay, the major active compounds that shown inhibitory activity against *B. pseudomallei* appeared at the lower end of the gel at the approximate molecular weight of less than 11 kDa as shown by silver stained gel (Fig. 4a, b). The active compounds could be small molecules as separate bands on the silver stain gel.

### Bio-control of *B. pseudomallei* in liquid medium

**Fig. 5:**
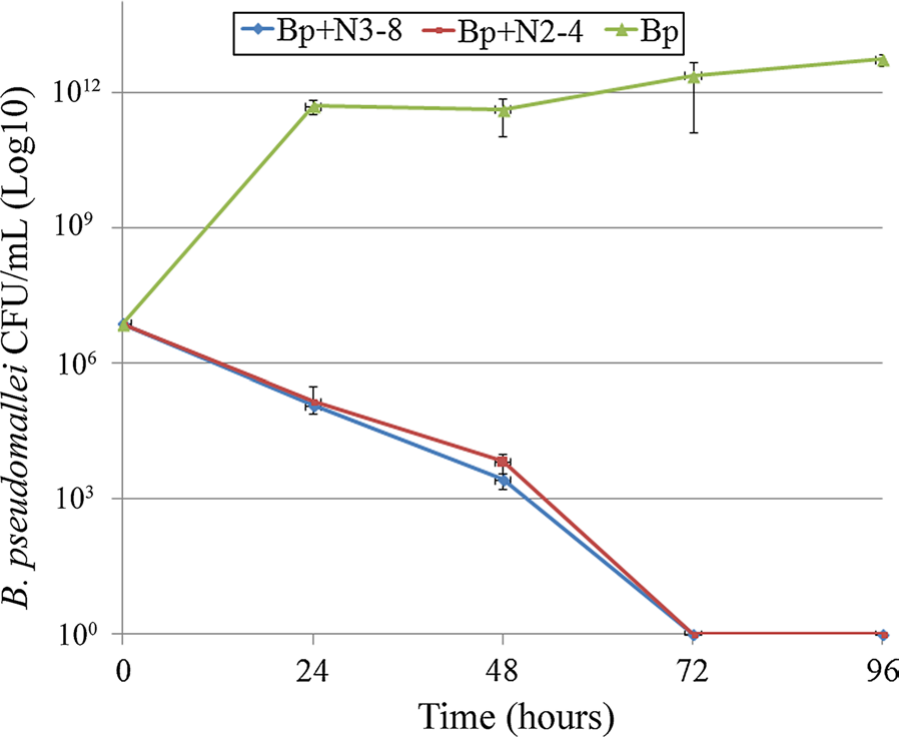
Bio-control by co-culture *B. pseudomallei* with *B. amyloliquefaciens*. The colony count of *B. pseudomallei* was plotted on the Y axis and time in hours on the X axis after co-culture with *B. amyloliquefaciens* isolates N2-4 (*red line*) and N3-8 (*blue line*) and the *green line* indicates the growth of un-treated *B. pseudomallei* as a control

N2-4 and N3-8 were co-cultured with 6.0 × 10^6^ CFU/ml of *B. pseudomallei* at a ratio of 1:100 in LB broth. The growth rate of *B. pseudomallei* appeared to be decreased by 5 log_10_ at 72 h after incubation when compared to the control growth (Fig. 5).

## Discussion

*Bacillus amyloliquefaciens* was reported to be a plant promoting bacterium that was associated with rhizospheres as they consume nutrients from the plant. Moreover, this group of bacteria could produce secondary metabolites to suppress plant pathogens and also produce compounds to promote plant growth (Idris et al. 2007). The completed genome of 3918 Kb of the FZB42 strain showed a total of 8.5% of genetic material to be associated with the synthesis function of the non-ribosomal secondary metabolites (Chen et al. 2007) and 3693 sequences encoded for proteins. *B. amyloliquefaciens* FZB42 produces several non-ribosomal peptide products such as lipopeptides; surfactin, fengycin, and bacillomycin D (Koumoutsi et al. 2004) that can inhibit *Fusarium oxysporum* and polyketides; bacillaene, difficidin and macrolactin that also inhibited *F. oxysporum* (Chen et al. 2006; Schneider et al. 2007). Moreover, the peptides plantazolicin A, B and cyclic peptides amylocyclicin produced from this bacterium were also reported to inhibit *B. subtilis* and other closely related bacteria (Kalyon et al. 2011; Scholz et al. 2014). Bacilysin a dipeptide product from *B. amyloliquefaciens* FZB42 was able to suppress growth of *E. amylovora* which is the causative agent of fire blight disease (Chen et al. 2009). In this current study, *B. amyloliquefaciens* N2-4 and N3-8 that were isolated from soil, negative for *B. pseudomallei,* could inhibit *B. pseudomallei* and a wide range of human pathogens.

The metabolites with antimicrobial activity secreted from *B. amyloliquefaciens* N2-4 and N3-8 isolates were produced at the early to mid-stationary phase (12–72 h) when cell density become increased and the activity decreased after 78 h of cultivation. This characteristic fits well to the secondary metabolites that can be induced by multifactor such as stress, starvation or environmental factors and also cell-to-cell communication or quorum sensing which use small peptides as inducer (Kleerebezem and Quadri 2001). Antimicrobial activity of compounds from *Bacillus* spp. from the Amazon river basin were also capable of producing compounds with antimicrobial activity at the exponential phase and reached their peak at the stationary phase (Motta et al. 2007). The antimicrobial activity of culture supernatant from N2-4 were decreased by heat, abolished when autoclaved and can be completely destroyed by proteolytic enzymes suggesting that the main active compounds against *B. pseudomallei* are proteins. For N3-8, the heat, autoclaving and proteolytic enzymes digestion could only partially decrease the activity. Therefore, the active compounds should compose of peptides that can be digested by proteolytic enzyme and other compounds that resist to proteolytic enzyme and high temperature conditions. As mentioned, *Bacillus* spp. were reported to produce both peptides and non-ribosomal peptide antibiotics that can inhibit peptidoglycan synthesis or cause pore formation using some specific molecules such as lipid II or the mannose phosphate transferase system (man**-**PTS) as a docking molecule (Cotter et al. 2013). Lipopeptides are metabolites that can function as a pore formation or emulsification on the target organism. Several peptides and bacteriocin-like substances from *Bacillus* spp. were reported to stable at a wide range of temperatures (30–80 °C) (Cao et al. 2011; Hammami et al. 2009; Sutyak et al. 2008). CLI proteins, for example, were stable up to 60 °C and lost activity after being autoclaved at 121 °C (Meng et al. 2012) and a peptide subtilosin was stable after heat at 100 °C (Sutyak et al. 2008). The antimicrobial substances from *B. amyloliquefaciens* N2-4 and N3-8 were stable up to 25–100 °C and some activity was decreased when they were heated above 80 °C. Moreover, the molecular weights of active fractions of precipitated proteins from both N2-4 and N3-8 were suspected to be less than 11 kDa. This information could indicate their metabolites to contain small peptides similar to bacteriocin antibiotics. Other compounds those were still active after proteolytic enzyme digestion could be the non-peptides group such as lipopeptides or polyketides. These antimicrobial substances were reported to resist a wide range of temperatures up to 100 °C (Pathak and Keharia 2014) and were also resistant to proteolytic enzyme digestion (Zhao et al. 2013).

The active compounds produced from both *B. amyloliquefaciens* N2-4 and N3-8 isolates were able to inhibit several strains of *B. pseudomallei* from both clinical and environmental sources including antibiotic resistant isolates. Moreover, they could inhibit other Gram-positive and Gram-negative bacteria such as *E. coli*, *S. aureus, E. faecium and C. difficile* as shown by the cross-streak method. Surprisingly, these antimicrobial compounds did not inhibit *B. thailandensis* the non-pathogenic bacteria that is closely related to *B. pseudomallei*. The difference in lipopolysaccharide (LPS) structure between the *B. pseudomallei* and *B. thailandensis* have been reported (Knirel et al. 1992; Perry et al. 1995) together with the difference in their genomes (Brett et al. 1998) may contribute to the difference in their susceptibility to the antimicrobial compounds. Most of *B. pseudomallei* in Thailand have the LPS genotype A (Tuanyok et al. 2012) and K96243 and 1026b strains with genotype A were susceptible to N2-4 and N3-8 metabolites, however, the SRM117 LPS O-side chain mutant was susceptible to N3-8 but not N2-4. Moreover, M6 and M10, which are biofilm mutants, were more susceptible to N3-8 than N2-4. The non-peptides compounds that are present in N3-8 but much less or none in the N2-4 as observed by heat and proteolytic stability test may be responsible for the differences when the culture supernatants were used to test for the spectrum of inhibition. Therefore, the precipitated proteins from N2-4 and N3-8 were prepared and tested with some pathogenic bacteria by agar well diffusion. Besides *S. pyogenes* and *M. catarrhalis* that showed equal inhibition, most of the test organisms were better inhibited by N3-8 than N2-4. Moreover, *V. parahaemolyticus* showed resistance to N2-4 but not N3-8. In general, most peptide antibiotics can inhibit bacteria in a narrow range or only closely related bacteria. Nevertheless, a bacteriocin-like substance of *Bacillus* spp. was reported to inhibit a broad-range of bacteria (Guo et al. 2012; Lee et al. 2001; Motta et al. 2007; Xie et al. 2009) that included *B. amyoliquefaciens* LBM5006 that inhibited *L. monocytogenes, B. cereus, Serratia marcescens, E. coli, P. aeruginosa, P. fluorescens, S. cholerasuis,* and *S. gallinarum* (Benitez et al. 2011). Similarly in this present study, the precipitated proteins from *B. amyloliquefaciens* N2-4 and N3-8 showed a broad range of inhibition. The metabolites of these two isolates should contain different compounds and the non-peptide metabolites in N3-8 may have a synergistic effect against these pathogens.

Several strains of *B. amyloliquefaciens* were studied to be used as bio-control agents such as the FZB42 strain that was dual-cultured with *E. amylovora,* both in vitro and in vivo, and showed inhibition activity in the growth of the pathogens (Chen et al. 2009). When *B. amyloliquefaciens* N2-4 and N3-8 were co-cultured with *B. pseudomallei,* they could decrease the growth of *B. pseudomallei* by 5 log_10_ in 72 h. The time of *B. pseudomallei* decrease was correlated with the time when the secondary metabolites from N2-4 and N3-8 were produced. Even though metabolites from N2-4 and N3-8 showed a broad spectrum of inhibition against both Gram-positive and Gram-negative pathogens, they did not affect *B. thailandensis,* a non-pathogenic bacterium from soil. Purification and characterization of both peptides and non-peptides from both isolates and the tests for their spectrum of inhibition may lead to a better knowledge to design a way for controlling *B. pseudomallei* in soil, and may also be extended to discover some important compounds to attack several problematic pathogens in the near future.

In conclusion, *B. amyloliquefaciens* N2-4 and N3-8 isolates obtained from soil can produce both peptides and non-peptide metabolites that can inhibit *B. pseudomallei* and a broad range of other pathogenic bacteria. After purification and characterization, the bacteria themselves or their metabolites could be used as bio-controls to reduce the pathogenic bacteria in soil of endemic *B. pseudomallei* areas. Moreover, if the compounds are novel and safe, they may be good candidates for the development of new drugs.

## Additional file

**Additional file 1.** Additional tables.

## Abbreviations

LB: Luria–Bertani
CFU: colony forming unit
MIC: minimum inhibitory concentration
MBC: minimum bactericidal concentration
CAZ: ceftazidime
Native-PAGE: native-polyacrylamide gel electrophoresis
EDTA: ethylenediamine tetrachloroacetic acid

## References

[CR1] Altschul SF, Madden TL, Schaffer AA, Zhang J, Zhang Z, Miller W, Lipman DJ (1997). Gapped blast and Psi-blast: a new generation of protein database search programs. Nucleic Acids Res.

[CR2] Barboza-Corona JE, Vazquez-Acosta H, Bideshi DK, Salcedo-Hernandez R (2007). Bacteriocin-like inhibitor substances produced by Mexican strains of *Bacillus Thuringiensis*. Arch Microbiol.

[CR3] Benitez L, Correa A, Daroit D, Brandelli A (2011). Antimicrobial activity of *Bacillus Amyloliquefaciens* Lbm 5006 is enhanced in the presence of *Escherichia Coli*. Curr Microbiol.

[CR4] Brett PJ, DeShazer D, Woods DE (1998). *Burkholderia Thailandensis* sp. nov., a *Burkholderia Pseudomallei*-like species. Int J Syst Bacteriol.

[CR5] Cao H, He S, Wei R, Diong M, Lu L (2011). *Bacillus Amyloliquefaciens* G1: a potential antagonistic bacterium against eel-pathogenic *Aeromonas Hydrophila*. Evid Based Complement Alternat Med.

[CR6] Chantratita N, Rholl DA, Sim B, Wuthiekanun V, Limmathurotsakul D, Amornchai P, Thanwisai A, Chua HH, Ooi WF, Holden MT, Day NP, Tan P, Schweizer HP, Peacock SJ (2011). Antimicrobial resistance to ceftazidime involving loss of penicillin-binding protein 3 in *Burkholderia Pseudomallei*. Proc Natl Acad Sci U S A.

[CR7] Chen XH, Vater J, Piel J, Franke P, Scholz R, Schneider K, Koumoutsi A, Hitzeroth G, Grammel N, Strittmatter AW, Gottschalk G, Sussmuth RD, Borriss R (2006). Structural and functional characterization of three polyketide synthase gene clusters in *Bacillus Amyloliquefaciens* Fzb42. J Bacteriol.

[CR8] Chen XH, Koumoutsi A, Scholz R, Eisenreich A, Schneider K, Heinemeyer I, Morgenstern B, Voss B, Hess WR, Reva O, Junge H, Voigt B, Jungblut PR, Vater J, Sussmuth R, Liesegang H, Strittmatter A, Gottschalk G, Borriss R (2007). Comparative analysis of the complete genome sequence of the plant growth-promoting bacterium *Bacillus Amyloliquefaciens* Fzb42. Nat Biotechnol.

[CR9] Chen XH, Scholz R, Borriss M, Junge H, Mogel G, Kunz S, Borriss R (2009). Difficidin and bacilysin produced by plant-associated *Bacillus Amyloliquefaciens* are efficient in controlling fire blight disease. J Biotechnol.

[CR10] Cheng AC, Currie BJ (2005). Melioidosis: epidemiology, pathophysiology, and management. Clin Microbiol Rev.

[CR11] Cotter PD, Ross RP, Hill C (2013). Bacteriocins - a viable alternative to antibiotics?. Nat Rev Microbiol.

[CR12] Errington J (2003). Regulation of endospore formation in *Bacillus Subtilis*. Nat Rev Microbiol.

[CR13] Ghribi D, Abdelkefi-Mesrati L, Mnif I, Kammoun R, Ayadi I, Saadaoui I, Maktouf S, Chaabouni-Ellouze S (2012). Investigation of antimicrobial activity and statistical optimization of *Bacillus Subtilis* Spb1 biosurfactant production in solid-state fermentation. J Biomed Biotechnol.

[CR14] Guo Y, Yu Z, Xie J, Zhang R (2012). Identification of a New *Bacillus Licheniformis* strain producing a bacteriocin-like substance. J Microbiol.

[CR15] Hammami I, Rhouma A, Jaouadi B, Rebai A, Nesme X (2009). Optimization and biochemical characterization of a bacteriocin from a newly isolated *Bacillus Subtilis* strain 14b for biocontrol of *Agrobacterium* spp. strains. Lett Appl Microbiol.

[CR16] Hemashenpagam N (2011). Purification of secondary metabolites from soil *Actinomycetes*. Int J Microbiol Res.

[CR17] Hoelzer K, Cummings KJ, Warnick LD, Schukken YH, Siler JD, Gröhn YT, Davis MA, Besser TE, Wiedmann M (2011). Agar disk diffusion and automated microbroth dilution produce similar antimicrobial susceptibility testing results for *Salmonella* serotypes newport, typhimurium, and 4,5,12:I-, but differ in economic cost. Foodborne Pathog Dis.

[CR18] Huang T-P, Tzeng DD-S, Wong ACL, Chen C-H, Lu K-M, Lee Y-H, Huang W-D, Hwang B-F, Tzeng K-C (2012). DNA polymorphisms and biocontrol of *Bacillus* antagonistic to citrus bacterial canker with indication of the interference of phyllosphere biofilms. PLoS ONE.

[CR19] Idris EE, Iglesias DJ, Talon M, Borriss R (2007). Tryptophan-dependent production of indole-3-acetic acid (Iaa) affects level of plant growth promotion by *Bacillus Amyloliquefaciens* Fzb42. Mol Plant Microbe Interact.

[CR20] Jamil B, Hasan F, Hameed A, Ahmed S (2007). Isolation of *Bacillus Subtilis* Mh-4 from soil and its potential of polypeptidic antibiotic production. Pak J Pharm Sci.

[CR21] Kalyon B, Helaly SE, Scholz R, Nachtigall J, Vater J, Borriss R, Sussmuth RD (2011). Plantazolicin a and B: structure elucidation of ribosomally synthesized thiazole/oxazole peptides from *Bacillus Amyloliquefaciens* Fzb42. Org Lett.

[CR22] Kleerebezem M, Quadri LE (2001). Peptide pheromone-dependent regulation of antimicrobial peptide production in gram-positive bacteria: a case of multicellular behavior. Peptides.

[CR23] Knirel YA, Paramonov NA, Shashkov AS, Kochetkov NK, Yarullin RG, Farber SM, Efremenko VI (1992). Structure of the Polysaccharide chains of *Pseudomonas Pseudomallei* lipopolysaccharides. Carbohydr Res.

[CR24] Koumoutsi A, Chen X-H, Henne A, Liesegang H, Hitzeroth G, Franke P, Vater J, Borriss R (2004). Structural and functional characterization of gene clusters directing nonribosomal synthesis of bioactive cyclic lipopeptides in *Bacillus Amyloliquefaciens* strain Fzb42. J Bacteriol.

[CR25] Lee KH, Jun KD, Kim WS, Paik HD (2001). Partial characterization of polyfermenticin scd, a newly identified bacteriocin of *Bacillus Polyfermenticus*. Lett Appl Microbiol.

[CR26] Meng QX, Jiang HH, Hanson LE, Hao JJ (2012). Characterizing a novel strain of *Bacillus Amyloliquefaciens* Bac03 for potential biological control application. J Appl Microbiol.

[CR27] Motta AS, Cannavan FS, Tsai SM, Brandelli A (2007). Characterization of a broad range antibacterial substance from a new *Bacillus* species isolated from amazon basin. Arch Microbiol.

[CR28] Naghili H, Tajik H, Mardani K, Razavi Rouhani SM, Ehsani A, Zare P (2013). Validation of drop plate technique for bacterial enumeration by parametric and nonparametric tests. Vet Res Forum.

[CR29] National Center for Biotechnology Information (2015) https://blast.ncbi.nlm.nih.gov/Blast.cgi. Accessed 1 Jul 2015

[CR30] Ngamsang R, Potisap C, Boonmee A, Lawongsa P, Chaianunporn T, Wongratanacheewin S, Rodrigues JL, Sermswan RW (2015). The contribution of soil physicochemical properties to the presence and genetic diversity of *Burkholderia Pseudomallei*. Southeast Asian J Trop Med Public Health.

[CR31] Palasatien S, Lertsirivorakul R, Royros P, Wongratanacheewin S, Sermswan RW (2008). Soil physicochemical properties related to the presence of *Burkholderia Pseudomallei*. Trans R Soc Trop Med Hyg.

[CR32] Pathak KV, Keharia H (2014). Application of extracellular lipopeptide biosurfactant produced by endophytic *Bacillus Subtilis* K1 isolated from aerial roots of banyan (*Ficus Benghalensis*) in microbially enhanced oil recovery (Meor). 3. Biotech.

[CR33] Perry MB, MacLean LL, Schollaardt T, Bryan LE, Ho M (1995). Structural Characterization of the lipopolysaccharide O antigens of *Burkholderia Pseudomallei*. Infect Immun.

[CR34] Ramli NS, Eng Guan C, Nathan S, Vadivelu J (2012). The effect of environmental conditions on biofilm formation of *Burkholderia Pseudomallei* clinical isolates. PLoS ONE.

[CR35] Sansinenea E, Ortiz A (2011). Secondary metabolites of soil *Bacillus* spp. Biotechnol Lett.

[CR36] Schägger H (2006). Tricine–sds-page. Nat Proc.

[CR37] Schneider K, Chen XH, Vater J, Franke P, Nicholson G, Borriss R, Sussmuth RD (2007). Macrolactin is the polyketide biosynthesis product of the Pks2 cluster of *Bacillus Amyloliquefaciens* Fzb42. J Nat Prod.

[CR38] Scholz R, Vater J, Budiharjo A, Wang Z, He Y, Dietel K, Schwecke T, Herfort S, Lasch P, Borriss R (2014). Amylocyclicin, a novel circular bacteriocin produced by *Bacillus Amyloliquefaciens* Fzb42. J Bacteriol.

[CR39] Sharma N, Kapoor G, Gautam N, Neopaney B (2009). Characterization of a partially purified bacteriocin of *Bacillus* sp Mtcc 43 isolated from rhizosphere of radish (*Raphanus Sativus*) & its application as a potential food biopreservative. J Sci Ind Res.

[CR40] Sopirala MM, Mangino JE, Gebreyes WA, Biller B, Bannerman T, Balada-Llasat JM, Pancholi P (2010). Synergy testing by etest, microdilution checkerboard, and time-kill methods for pan-drug-resistant *Acinetobacter Baumannii*. Antimicrob Agents Chemother.

[CR41] Sutyak KE, Wirawan RE, Aroutcheva AA, Chikindas ML (2008). Isolation of the *Bacillus Subtilis* antimicrobial peptide subtilosin from the dairy product-derived *Bacillus Amyloliquefaciens*. J Appl Microbiol.

[CR42] Travers RS, Martin PAW, Reichelderfer CF (1987). selective process for efficient isolation of soil *Bacillus* spp. Appl Environ Microbiol.

[CR43] Tuanyok A, Stone JK, Mayo M, Kaestli M, Gruendike J, Georgia S, Warrington S, Mullins T, Allender CJ, Wagner DM, Chantratita N, Peacock SJ, Currie BJ, Keim P (2012). The genetic and molecular basis of O-antigenic diversity in *Burkholderia Pseudomallei* lipopolysaccharide. PLoS Negl Trop Dis.

[CR44] Umer S, Tekewe A, Kebede N (2013). Antidiarrhoeal and Antimicrobial activity of *Calpurnia Aurea* leaf extract. BMC Complement Altern Med.

[CR45] Wiersinga WJ, Currie BJ, Peacock SJ (2012). Melioidosis. N Engl J Med.

[CR46] Xie J, Zhang R, Shang C, Guo Y (2009). Isolation and characterization of a bacteriocin produced by an isolated *Bacillus Subtilis* Lfb112 that exhibits antimicrobial activity against domestic animal pathogens. Afr J Biotechnol.

[CR47] Zhao P, Quan C, Jin L, Wang L, Wang J, Fan S (2013). Effects of critical medium components on the production of antifungal lipopeptides from *Bacillus Amyloliquefaciens* Q-426 exhibiting excellent biosurfactant properties. World J Microbiol Biotechnol.

