# Supplementary material for: Secondary metabolites from Bacillus amyloliquefaciens isolated from soil can kill Burkholderia pseudomallei
Source: AMB Express. 2017 Jan 4;7:16. doi: 10.1186/s13568-016-0302-0 (PMC5209304; doi:10.1186/s13568-016-0302-0)
Supplement: Supplementary file 1 — Additional file 1. Additional tables. [file 13568_2016_302_MOESM1_ESM.docx]

**Supplementary materials**

**AMB express**

**Secondary metabolites from *Bacillus amyloliquefaciens* isolated from soil can kill**

***Burkholderia pseudomallei***

Patcharaporn Boottanun^1^, Chotima Potisap^1^, Julian G. Hurdle^2^, Rasana W Sermswan^1^ ^🖂^

^1^Melioidosis Research Center and Department of Biochemistry, Faculty of Medicine, Khon Kaen University, Khon Kaen, Thailand, 40002

^2^Center for Infectious and Inflammatory diseases, Institute of Biosciences and Technology, Texas A&M University, Texas 77030, USA

**Corresponding author**

Rasana W Sermswan

Department of Biochemistry and Melioidosis Research Center

123 Mitraparb Rd, Muang district, Khon Kaen Province, Thailand, 40002

E-mail: [rasana@kku.ac.th](mailto:rasana@kku.ac.th)

Tel/Fax: 66-43-363265,

Mobile: 66-89-862-2606

**Table S1.** Bacterial strains

| **Bacterial strains** | |
| --- | --- |
| ***Burkholderia pseudomallei*** (WDCM 1130)  **Clinical isolates**  P37  NF10/38  EPKKU11  EPKKU12  EPKKU27  EPKKU32  EPKKU15  EPMK31  EPUT222  EPUT239  EPNL7  EPSK15  EPSN41  K96243  H777  1026b  **Environmental isolates**  ZSK1  ZSK4  ZSK5  ZSK6  ZSK7  ZSK9  **Ceftazidime resistant isolates**  365A  316C  979B  EPMN34  EPMN159  **Mutant strains**  M6  M10  SRM117  MM35  SR1015  ***Burkholderia thailandensis*** (WDCM 1130)  20-3  47  49-1 | ***Burkholderia thailandensis*** (WDCM 1130)  50-2  51-3  58  59-1  89-2  MBPE159  MBPE164  MBPE170  UE5  **Gram-positive**  *Bacillus amyloliquefaciens* N2-4 (WDCM 1130)  *Bacillus amyloliquefaciens* N3-8 (WDCM 1130)  *Clostidium difficile* (ATCC:BAA-1875)  *Corynebacterium diphtheriae*  *Streptococcus pneumoniae*  *Streptococcus pyogenes*  *Staphylococcus aureus* newman (ATCC:25904)  *Staphylococcus aureus* JE2 (NR-46553)  *Enterococcus feacalis*  *Enterococcus feacium*  **Gram-negative**  *Acinetobacter baumannii*  *Citrobacter freundii*  *Escherichia coli* *Escherichia coli* K12 (ATCC:PTA-7555) *Escherichia coli* Tolc  *Enterobacter cloacae*  *Enterobacter cloacae* (ATCC:BAA1443)  *Klebsiella pneumonia*  *Klebsiella pneumonia* (ATCC:13883)  *Moraxella catarrhalis*  *Proteus vulgaris*  *Pseudomonas aeruginosa*  *Pseudomonas aeruginosa* PAO1 (ATCC:BAA­47)  *Salmonella* group D  *Shigella* group D  *Stenotrophomonas maltophilia*  *Vibrio parahaemolyticus* |

**Table S2.** Thermal stability test of antimicrobial activity against *B. pseudomallei* of the culture supernatants from *B. amyloliquefaciens* N2-4 and N3-8

| **Temperature (°C)**  **15 minutes** | **Inhibition zone (Ø mm) against *B. pseudomallei*** | |
| --- | --- | --- |
|  | **N2-4 supernatant** | **N3-8 supernatant** |
| 25 | 20 | 25 |
| 37 | 20 | 25 |
| 40 | 19 | 22 |
| 50 | 22 | 19 |
| 60 | 21 | 18  18 |
| 70 | 22 |  |
| 80 | 18 | 22 |
| 90 | 17 | 22 |
| 100 | 17 | 20 |
| 121 | 0 | 12 |
| Ceftazidime | 27 | 25 |

**Table S3.** Proteolytic enzyme susceptibility test of culture supernatants from *B. amyloliquefaciens* N2-4 and N3-8

| **Enzymes treated** | **Inhibition zone (Ø mm) against *B. pseudomallei*** | |
| --- | --- | --- |
|  | **N2-4 supernatant** | **N3-8 supernatant** |
| Papain treated | 12 | 14 |
| Control supernatant | 17 | 20 |
| Trypsin treated | 0 | 15 |
| Control supernatant | 18 | 21 |
| Pepsin treated | 11 | 12 |
| Control supernatant | 17 | 20 |
| Proteinase K treated | 0 | 14 |
| Control supernatant | 14 | 17 |

**Table S4.** The antimicrobial activity of *B. amyloliquefaciens* N2-4 and N3-8 isolates against pathogenic bacteria observed by cross streak method

| **Bacterial indicators** | **Inhibitory activity** | |
| --- | --- | --- |
|  | **N2-4** | **N3-8** |
| *S. aureus* newman | **++++** | **++++** |
| *S. aureus* JE2 | **++++** | **++++** |
| *S. pyogenes* | **++** | **+** |
| *E. fecalis* | **+++** | **+++** |
| *E. faecium*  *B. pseudomallei* P37 | **++++**  **+** | **++++**  **+** |
| *E. coli* K12 | **+++** | **+++** |
| *E. coli* Tolc | **++** | **+++** |
| *E. cloacae* 1443 | **+** | **+** |
| *A. baumannii* | **+** | **+** |
| *K. pnueminiae* 13883 | **+** | **+** |
| *P. aeroginosa* PAO1 | **+** | **+** |
| *C. difficile* 1875 | **++++** | **+++** |

Symbols: ++++ for the highest, +++ for moderate, ++ for a few, + for low activity and – for no inhibition.
